# Diabetes Self-Management Education and Training Among Privately Insured Persons with Newly Diagnosed Diabetes — United States, 2011–2012

**Published:** 2014-11-21

**Authors:** Rui Li, Sundar S. Shrestha, Ruth Lipman, Nilka R. Burrows, Leslie E. Kolb, Stephanie Rutledge

**Affiliations:** 1 Division of Diabetes Translation, National Center for Chronic Disease Prevention and Health Promotion, CDC; 2 American Association of Diabetes Educators

Diabetes is a complex chronic disease that requires active involvement of patients in its management (1). Diabetes self-management education and training (DSMT), “the ongoing process of facilitating the knowledge, skill, and ability necessary for prediabetes and diabetes self-care,” is an important component of integrated diabetes care (2). It is an intervention in which patients learn about diabetes and how to implement the self-management that is imperative to control the disease. The curriculum of DSMT often includes the diabetes disease process and treatment options; healthy lifestyle; blood glucose monitoring; preventing, detecting and treating diabetes complications; and developing personalized strategies for decision making (2). The American Diabetes Association recommends providing DSMT to those with newly diagnosed diabetes (1), because data suggest that when diabetes is first diagnosed is the time when patients are most receptive to such engagement (3). However, little is known about the proportion of persons with newly diagnosed diabetes participating in DSMT. CDC analyzed data from the Marketscan Commercial Claims and Encounters database (Truven Health Analytics) for the period 2009–2012 to estimate the claim-based proportion of privately insured adults (aged 18–64 years) with newly diagnosed diabetes who participated in DSMT during the first year after diagnosis. During 2011–2012, an estimated 6.8% of privately insured, newly diagnosed adults participated in DSMT during the first year after diagnosis of diabetes. These data suggest that there is a large gap between the recommended guideline and current practice, and that there is both an opportunity and a need to enhance rates of DSMT participation among persons newly diagnosed with diabetes.

The Marketscan private insurance database includes data from both the employer and health plans that cover active employees, their spouses, and dependents. The database contains fully adjudicated and paid claims for millions of enrollees (e.g., approximately 52 million in 2011), including patient-level enrollment and inpatient, outpatient, and prescription drug claims. Persons were assigned a diagnosis of diabetes using the following algorithm: 1) having at least two outpatient claims ≥30 days apart coded for diabetes as a primary or secondary diagnosis (*International Classification of Diseases, Ninth Revision, Clinical Modification* codes 250x), 2) having received prescriptions for diabetes medications, either oral agents or insulin (therapeutic class codes 172–174), or 3) having at least one inpatient admission with diabetes as a primary or secondary diagnosis. Persons were classified as being newly diagnosed if they had diabetes in 2011 but not in 2010 and 2009. For inclusion in the study, persons were required to be continuously enrolled in 2009, 2010, and 2011 to minimize misclassification of persons with existing diabetes as newly diagnosed. Furthermore, they had to be continually enrolled for at least 12 months post-diagnosis to consistently capture DSMT participation during the first year after diagnosis. They were also required to have prescription drug coverage to ensure the accurate classification of antiglycemic medication use.

DSMT participation was defined as having filed at least one DSMT claim (G0108, G0109, S9140, S9141, S9145, S9455, S9460, and S9465) within 12 months after diagnosis of diabetes.* DSMT participation was estimated overall and for subgroups by age, sex, oral diabetes medication prescription, insulin prescription, insurance type (fee-for-service or capitated health plan), metropolitan statistical area, and region of residence, using multivariate logistic regression. Predicted margins were reported as adjusted rates of DSMT participation in these subgroups, adjusting simultaneously for the other covariates. The difference in adjusted rates of DSMT participation between subgroups was tested using t-tests; results were considered statistically significant if p<0.05.

A total of 95,555 persons with newly diagnosed diabetes were identified. Among them, 25.6% were not prescribed any antiglycemic medications, and 6.8% were prescribed insulin (with or without oral medication) (Table 1). During 2011–2012, 6.8% of persons with newly diagnosed diabetes participated in DSMT within 12 months of diagnosis.

The adjusted rates of participation in DSMT were slightly higher among older (aged 45–64 years) compared with younger adults (aged 18–44 years) (7.2% versus 5.9%, p<0.001); those prescribed insulin for glycemic control compared with those prescribed oral agents only (14.2% versus 6.7%, p<0.001) or not prescribed any antiglycemic medication (14.2% versus 5.1%, p<0.001); those enrolled in fee-for-service health plans compared with those in capitated health plans (7.0% versus 6.0%, p<0.001); those residing in a metropolitan statistical area compared with those outside (7.1% versus 5.5%, p<0.001); and those residing in the North Central region (9.2%) compared with those residing in other regions (5.7%–6.9%, p<0.001 for each) (Table 2). For each subgroup, the adjusted rate of participating in a DSMT ranged from 5.1% to 14.2%.

## Discussion

DSMT helps patients to improve glycemic control, which could reduce the risk for diabetes complications, hospitalizations, and health care costs (4–6). The findings in this report indicate that DSMT was substantially underused among persons with newly diagnosed diabetes even in an insured population with private health insurance. Fewer than 7% of persons received DSMT within 1 year after diagnosis with diabetes. Although there were differences in the rates of DSMT participation across subgroups, no subgroup of persons with newly diagnosed diabetes reached even a 15% participation rate.

In this report, DSMT classification was based on actual claims for DSMT received in the health care setting. Another analysis using cross-sectional commercial and Medicare claims-based databases also reported low rates of participation in DSMT and nutrition therapy in all enrollees with diagnosed diabetes (7% among those with private insurance and 4% among those with Medicare coverage) (6). Furthermore, the age-adjusted percentage of adults aged ≥18 years with diagnosed diabetes reported ever having attended a diabetes education class was 57.4% in 2010 (7), falling short of the *Healthy People 2020* objective D-14: increase the proportion of persons with diagnosed diabetes who receive formal diabetes education to 62.5%.†

What is already known on this topic?

Diabetes self-management education and training (DSMT) is an important part of clinical management of diabetes that helps persons with diabetes stay healthy. The American Diabetes Association recommends persons with diabetes receiving DSMT at diagnosis and as needed thereafter. Diabetes education is associated with increased use of primary and preventive services and lower use of acute, inpatient hospital services.

What is added by this report?

Among persons aged 18–64 years with newly diagnosed diabetes who had private insurance coverage, the rate of participation in DSMT during the first year after diagnosis was very low (6.8%). The rate was <15% among all subgroups examined.

What are the implications for public health practice?

Health system level interventions such as improving access to DSMT, along with personal level interventions such as behavioral change strategies, might be considered to increase the rate of DSMT participation among persons with newly diagnosed diabetes.

Lack of insurance coverage has previously been identified as a barrier to DSMT participation (8). Based on previous research, 44 states§ required private insurance to cover DSMT, but many plans still did not cover it, and many others required a copayment (9). An additional health system barrier might be the requirement for physician referral for DSMT (8). There are also individual-level barriers, such as personal perceptions about diabetes, avoidance behaviors, and lack of awareness that DSMT exists (8).

Low DSMT participation among persons with newly diagnosed diabetes is a concern. Although some persons might have participated in medical nutrition therapy, from which they receive nutrition recommendations and interventions, others might have limited knowledge about the dietary aspects of diabetes management (1). For those not prescribed medication for glycemic management, failure to participate in DSMT could mean that their diabetes remains essentially untreated. For those prescribed insulin, lack of participation in DSMT could reduce the likelihood of adequate blood glucose management (1). The American Medical Association–convened Physician Consortium for Performance Improvement and the National Committee for Quality Assurance have proposed additional quality indicators for diabetes care, including rates of referral to DSMT for patients newly diagnosed with diabetes and rates of referral to DSMT for patients with newly prescribed insulin.¶

The findings in this report are subject to at least four limitations. First, the study population was limited to persons aged 18–64 years who were covered by employer-provided health insurance in the Marketscan database and had continuous coverage for ≥3 years. Therefore, these findings might not be generalizable to other populations, such as those aged ≥65 years and persons with other types of health insurance coverage or without health insurance (6). Second, participation in DSMT was defined as having had at least one claim filed for DSMT. Some persons might have received DSMT that was not covered by insurance (e.g., through a worksite wellness program). Third, multiple DSMT visits during the first year after diagnosis of diabetes are often recommended in clinical guidelines; whether or not persons participating in DSMT completed all the recommended hours is unknown. Finally, claims data might include some misclassification and misreporting, and the claim-based algorithm to define diabetes patients might have underestimated the number of persons with diagnosed diabetes. However, studies have shown that claims-based data adequately identify most persons with diagnosed diabetes (10).

The finding of low rates of participation in DSMT among privately insured adults with newly diagnosed diabetes underscores the need to identify specific barriers to access and participation in DSMT along with strategies to overcome these barriers. CDC is working to achieve *Healthy People 2020* objective D-14. In 2013, CDC administered funds to state health departments to implement DSMT strategies through the 5-year cooperative agreement: State Public Health Actions to Prevent and Control Diabetes, Heart Disease, Obesity, and Associated Risk Factors and Promote School Health. The DSMT strategy under this agreement focuses on increasing use by persons with diabetes of DSMT programs recognized by the American Diabetes Association or accredited by the American Association of Diabetes Educators, through increased access, physician referrals, and reimbursement.

## Figures and Tables

**TABLE 1:** Selected characteristics of persons enrolled in a study assessing participation in diabetes self-management education and training* — United States, 2011–2012

Characteristic	%
**Age group (yrs)**	
18–44	29.0
45–64	71.0
**Female**	53.4
**Diabetes treatment**	
Insulin (with or without oral antiglycemic medication)	6.8
Oral antiglycemic medication only	67.6
Without antiglycemic medication	25.6
**Health plan**	
Fee-for-service	81.2
Capitated	18.8
**Living in an MSA**	84.1
**U.S. Census region**	
Northeast	11.7
North Central	23.3
South	47.5
West	17.5

**Abbreviation:** MSA = metropolitan statistical area.

**Source:** Marketscan Commercial Claims and Encounters database (Truven Health Analytics).

* Enrollees 1) were adults aged 18–64 years with diabetes newly diagnosed in 2011; 2) were continuously enrolled in a private health plan in 2009, 2010, and 2011, and during the year after diagnosis of diabetes; and 3) had prescription drug coverage.

**TABLE 2:** Adjusted percentage* of study enrollees (N = 95,555)† participating in diabetes self-management education and training within 1 year after being diagnosed with diabetes, by selected characteristics — United States, 2011–2012

Characteristic	%
**Overall**	**6.8**
**Age group (yrs)**	
18–44	5.9
45–64	7.2
**Sex**	
Male	6.8
Female	6.8§
**Diabetes treatment**	
Insulin (with or without oral antiglycemic medication)	14.2
Oral antiglycemic medication only	6.7
Without antiglycemic medication	5.1
**Health plan**	
Fee-for-service	7.0
Capitated	6.0
**Place of living**	
MSA	7.1
Non-MSA	5.5
**U.S. Census region**	
Northeast	6.9
North Central	9.2
South	5.7
West	6.5

**Abbreviation:** MSA = metropolitan statistical area.

**Source:** Marketscan Commercial Claims and Encounters database (Truven Health Analytics).

* Predicted margins adjusted simultaneously for age, sex, medication use, insurance type, MSA, and U.S. Census region. Comparison of rates between subgroups and the reference group are all statistically significant (p<0.001), except those designated as not significant (p>0.05). Reference groups: aged 18–44 years, male, insulin prescription, fee-for-service plan, MSA, and North Central region.

† Enrollees 1) were adults aged 18–64 years with diabetes newly diagnosed in 2011; 2) were continuously enrolled in a private health plan in 2009, 2010, and 2011, and during the year after diagnosis of diabetes; and 3) had prescription drug coverage.

§ Not statistically significant (p>0.05).

## Footnotes

* Additional information available at http://www.diabeteseducator.org/export/sites/aade/_resources/pdf/reimbursement_tips_2009.pdf.

† Additional information available at https://www.healthypeople.gov/2020/topics-objectives/topic/diabetes/objectives.

§ Additional information available at http://www.ncsl.org/research/health/diabetes-health-coverage-state-laws-and-programs.aspx.

¶ Additional information available at http://www.ama-assn.org/ama1/pub/upload/mm/pcpi/diabetesset.pdf.
